# The NICE ADHD health technology assessment: A review and critique

**DOI:** 10.1186/1753-2000-2-1

**Published:** 2008-01-15

**Authors:** Michael Schlander

**Affiliations:** 1 Institute for Innovation & Valuation in Health Care (InnoVal^HC^), Rathausplatz 12-14, D-65760 Eschborn, Germany; 2 Department of Public Health, Social and Preventive Medicine, Mannheim Medical Faculty, University of Heidelberg, Ludolf-Krehl-Strasse 7-11, D-68167 Mannheim, Germany; 3 University of Applied Economic Sciences Ludwigshafen, Ernst-Boehe-Strasse 4, D-67059 Ludwigshafen, Germany

## Abstract

**Background:**

Health technology assessments (HTAs) by the National Institute for Health and Clinical Excellence (NICE) enjoy high levels of international attention. The present analysis addresses NICE's appraisal of methylphenidate, atomoxetine and dexamphetamine for attention-deficit/hyperactivity disorder (ADHD) in children and adolescents, published in March 2006.

**Methods:**

A qualitative study of NICE Technology Appraisal No. 98 was done focusing on the >600-page technology assessment report, which aimed at evaluating ADHD treatment strategies by a clinical effectiveness review and an economic analysis using meta-analytical techniques and a cost-effectiveness model.

**Results:**

The technology assessment was unable to differentiate between the various drugs in terms of efficacy, and its economic model was ultimately driven by cost differences. While the assessment concluded that the economic model "clearly identified an optimal treatment strategy" with first-line dexamphetamine, the NICE appraisal committee subsequently found it impossible to distinguish between the different strategies on grounds of cost-effectiveness. Analyzing the assessment reveals gaps and inconsistencies concerning data selection (ultimately relying on a small number of short-term studies only), data synthesis (pooling of heterogeneous study designs and clinical endpoints), and economic model structure (identifying double-counting of nonresponders as a likely source of bias, alongside further methodological anomalies).

**Conclusion:**

Many conclusions of the NICE technology assessment rest on shaky grounds. There remains a need for a new, state-of-the-art systematic review of ADHD treatment strategies including economic evaluation, which ideally should address outcomes beyond children's health-related quality of life, such as long-term sequelae of the disorder and caregiver burden.

## Background

Health care policy makers and clinicians increasingly seek evidence-based guidance on how to provide mental health services effectively and efficiently. Systematic reviews have come to be accepted to produce the best estimates of clinical efficacy and effectiveness, thus constituting a cornerstone of policy analysis and cost-effectiveness evaluation [1]. Founded in 1999, the London-based National Institute for Health and Clinical Excellence (NICE) has quickly established itself as a leading agency conducting health technology assessments (HTAs) including economic evaluation. A review team of the World Health Organization observed that its "published technology appraisals are already being used as international benchmarks" [2], beyond NICE's primary remit to provide guidance for the National Health Service in England and Wales.

In March 2006, NICE published guidance on the use of methylphenidate, atomoxetine and dexamphetamine for attention-deficit/hyperactivity disorder (ADHD) in children and adolescents (NICE Technology Appraisal No. 98 [3]). This guidance was based on a >600-page technology assessment report, which had been produced by a team of ten experts, including one clinical specialist who provided input and comments [4].

In the United States, NICE guidelines are routinely referenced by the National Guideline Clearinghouse, an initiative of the Agency for Healthcare Research and Quality (AHRQ). NICE technology assessments and appraisals are easily accessible through its website, which receives more than one million hits per month from the United States alone [5]. The NICE assessment of ADHD treatment strategies thus might have great influence on future treatment practices beyond England and Wales, notably including the United States where ADHD now is the most commonly diagnosed behavioral disorder in children, with approximately 4.4 million diagnosed and 2.5 million taking medication for the disorder in the age group 4 – 17 years [6]. Therefore a critical appraisal of NICE Technology Appraisal No. 98 will be of interest to mental health care policy makers and clinicians.

## Methods

A qualitative study was done of NICE Technology Appraisal No. 98, "Methylphenidate, atomoxetine and dexamfetamine for attention deficit hyperactivity disorder (ADHD) in children and adolescents (Review of Technology Appraisal 13)" [3]. The study focused on policy-relevant aspects and had descriptive, explorative, and explanatory elements.

Its initial phase consisted of defining a theoretical framework for analysis. This included a description of NICE technology appraisal processes, which fell in a period of substantial upgrade and definition of "reference case" analysis by NICE [7,8]. During this phase, a thematic framework was defined, comprising use of the "accountability for reasonableness" concept as a process benchmark [9,10], a critique of the technology assessment report underlying the appraisal, as well as a review of the clinical and economic literature on attention-deficit/hyperactivity disorder [11].

Its second phase comprised data collection employing a number of closely related strategies, including retrieval and analysis of documents related to the ADHD appraisal which were posted on the NICE website. Scientific articles cited in these documents were obtained for analysis. This was supplemented by literature searches (using the PubMed and, via EBSCO host services, the Business Source Elite databases as well as Google Scholar) for articles on ADHD diagnosis, treatment, compliance, cost, and cost-effectiveness, which were complemented by a search for relevant abstracts presented at international meetings in the fields of psychiatry and health economics. Documents were indexed using categories including study type, product tested, and subject matter (e.g., "treatment compliance") for further analysis and interpretation.

The analysis reported here is part of this more comprehensive study of NICE appraisal processes by the same author [11], and it is focused on the underlying Technology Assessment Report [4]. The purpose of the present paper is to shed light on the validity of the conclusions offered by NICE; it should be emphasized that it is not intended to assign responsibility for any identified problems to particular actors (such as NICE, its committees, or the assessment team). Unless specified otherwise, the following citations will refer to the Technology Assessment Report ("TAR" [4]), which was subsequently published as a full paper in the *Health Technology Assessment *monograph series of the NHS R&D HTA Programme [12], apparently unchanged.

## Results

The various products evaluated by NICE are summarized in Table 1. The scope of the assessment [13] and its final protocol [14] specified that these products should be compared to placebo and usual care. Outcomes should include the incidence and severity of core symptoms, problem behaviors, educational performance, measures of depression and/or anxiety, measures of conduct/oppositional-defiant-disorder-related outcomes, adverse events, and quality of life. A recommendation was also included to consider the impact of co-morbid disorders, quality of life of family members, and optimal duration of treatment, "where the evidence permits". The scope effectively excluded an evaluation of non-drug treatment and of ADHD in adults. Also alternative treatments were not reviewed [15].

**Table 1:** Products evaluated by NICE

**Trade Name (England)**	**Active Ingredient**	**Formulation**	**Cost/Daily Dose**^1^	**Assumed Total Daily Dose**	**Daily Dosage Schedule**
Dexedrine	Dex amphetamine	Short-acting	₤ 0.43	20 mg/d	2 (-3) times
Ritalin	Methyl phenidate	Short-acting	₤ 0.56	30 mg/d	3 (2–4) times
Equasym	Methyl phenidate	Short-acting	₤ 0.53	30 mg/d	3 (2–4) times
Concerta XL	Methyl phenidate	Long-acting (~12 h)	₤ 1.23	36 mg/d	1 time
Equasym XL	Methyl phenidate	Long-acting (~8 h)	₤ 1.17	30 mg/d	1 time
Strattera	Atomoxetine	Long-acting	₤ 1.95	Irrelevant (flat pricing)	1 (-2) times

^1^Acquisition costs of the National Health Service (NHS) in England (net prices, excluding VAT and not accounting for negotiated procurement discounts in some settings), from British National Formulary 51, March 2006; note that individual doses and thus costs may vary. Assumed average doses should not be confused with dose recommendations.

The Technology Assessment Report (TAR), comprising 605 pages with 13 appendices, included a systematic review of the evidence and a statistical data synthesis using mixed treatment comparison (MTC) techniques, a review of submissions by manufacturers, and an economic evaluation model. Main conclusions of the TAR were that "(i) drug therapy seems to be superior to no drug therapy; (ii) no significant differences between the various drugs in terms of efficacy or side effects were found – mainly due to *lack of evidence*; (iii) the additional benefits from behavioural therapy (in combination with drug therapy) are uncertain" and: "Given the lack of evidence for any differences in effectiveness between the drugs, the [economic] model tends to be driven by drug cost, which differ considerably" (TAR, p. 20). More specifically, it was asserted that "for a decision taken now, with current available data, *the results of the economic model clearly identify an optimal treatment strategy*" (TAR, p. 261; italics added) and that "this analysis showed that a treatment strategy of 1st line dexamphetamine, followed by 2nd line methylphenidate immediate-release for treatment failures, followed by 3rd line atomoxetine for repeat treatment failures was optimal" (TAR, p. 260).

Remarkably the NICE Appraisal Committee did not uphold these "clear conclusions", issuing guidance that was based on the assumption that it was "not possible to distinguish between the different [treatment] strategies on the grounds of cost-effectiveness" [3].

Analysis of the TAR reveals a number of methodological issues, which collectively leave the assessment open to critique concerning all four essential components of a review question [16], namely the population studied (e.g., exclusion of adults and failure to address the impact of coexisting conditions), the choice of interventions (e.g., exclusion of psychosocial treatment), the clinical and economic outcomes criteria used, as well as the study designs and selection criteria. The following critique will concentrate on key issues concerning data selection and synthesis as well as the model developed for economic evaluation.

### Data selection for assessment

Departing from the assessment protocol [14], literature searches for assessment did not include "abstracts, ... conference proceedings, ... and other grey literature etc." (see final protocol, pp. 2f. [14]; cf. also TAR, p. 178), thus excluding relevant cost-effectiveness analyses in the public domain at the time of assessment [17-20]. This notwithstanding, it was claimed that "this review presents a comprehensive overview of existing economic evaluations of methylphenidate, atomoxetine and dexamphetamine for children and adolescents with ADHD" (TAR, p. 266). Further search anomalies include the overlooking of at least two clinical studies meeting the specified inclusion criteria [21,22].

For assessment, studies had to have a minimum duration of three weeks because "the literature suggests that three weeks is the minimum duration for therapeutic trials" to assess "the impact on the social adjustment of the child" (TAR, p. 44), citing the DSM-IV diagnostic manual (TAR, p. 45). The rationale offered was that "the effect of medication on behaviour is often (not always) apparent immediately, but the impact on the social adjustment of the child my well not be apparent in the first days of therapy (final assessment protocol [14], pp. 3ff., and TAR, p. 45). To make sense out of this reasoning, one would expect a minimum *treatment *duration of three weeks. However, a minimum *study *duration of three weeks was applied as inclusion criterion. As a consequence, more than one third of the 64 trials included in the clinical effectiveness review were very-short-term crossover studies with treatment periods of one week or less, some of which had been conducted without washout phases between treatment periods (TAR, pp. 51–163) [11].

The observation that the choice of outcomes measures reflects a critical design choice for analysis (TAR, p. 178) was not followed by a review of the literature on measurement instruments [23,24]. Although social adjustment of patients was implied to be the outcome of interest (TAR, p. 45), clinical effect measures reflecting functional impairment were discarded from analysis (TAR, p. 46). Instead, clinical global impressions (CGI scores) were used "as a proxy of quality of life" (TAR, pp. 16 and 48), and CGI-I (improvement) subscores were selected as the primary endpoint informing economic evaluation. In this respect, the economic analysis deviated from the clinical effectiveness review that had included measures of hyperactivity and comprised a total of 64 randomized clinical trials (RCTs) plus the MTA study [25,26]. This decision was motivated by the desire to compute quality-adjusted life years (QALYs) as effectiveness measure for economic modeling (TAR, pp. 224f.). Since this maneuver left only five RCTs with treatment durations from three to eight weeks for modeling, and none of those included dexamphetamine, a study previously excluded for inadequate data presentation was secondarily added in order to have *any *data on dexamphetamine for economic analysis (TAR, pp. 225f. and p. 338). Given well-documented gender differences in ADHD [27], which include clinical response to methylphenidate [28], and the fact that the disorder is most often diagnosed in boys [29], it is noteworthy that this three-weeks cross-over study had reported on 32 girls [30]. The assessment did not offer a discussion of this peculiarity. On this basis, after eliminating consideration of the role of concomitant psychosocial interventions, 19 alternative treatment "strategies" (in fact, product sequences) were modeled (TAR, p. 221).

Another important gap occurred in relation to atomoxetine. First, one out of two state-of-the-art RCTs comparing atomoxetine and long-acting methylphenidate was overlooked [22,31]. These studies concurred suggesting lower or at best equal efficacy of atomoxetine [22,31-33]. Second, two analyses were not considered that provided effect size estimates for long-acting stimulants (0.95 – 1.02) and nonstimulant medications (0.44 – 0.62), using core symptom improvement as effect measure [34-37]. These findings had been interpreted by their authors as "substantial and significant differences in efficacy" [37].

### Data synthesis

In an attempt to overcome the limitations of the remaining database, the NICE assessment relied on advanced mixed-treatment comparison techniques for quantitative meta-analysis of response rates, which were subsequently transformed into QALY gains. For QALY computation, quality weights were derived from utility studies that had used health state descriptions, which did neither correspond to the CGI criteria nor to any of the other clinical endpoints secondarily added (cf. TAR, pp. 359ff.). This approach was pursued despite explicit recognition that "the validity of these measures depends on the content and style of the vignette used to describe each health state" (TAR, p. 181).

In order to broaden the database of six RCTs, data derived from different clinical effect measures were subsequently pooled for "sensitivity analyses"; data synthesis comprised heterogeneously defined "response rates" based on (in addition to the CGI-I subscores) CGI-S ratings as well SNAP-IV and ADHD-RS scores (TAR, p. 254), while the most widely used measures of clinical efficacy in ADHD trials, the Conners Rating Scales [23,24], remained excluded from economic modeling (TAR, p. 224) despite their well-documented psychometric properties [24]. These narrow-band symptom scales (i.e., the SNAP-IV and ADHD-RS [23,24]) were erroneously regarded as "disease-specific instruments [measuring] health-related quality of life in children" (TAR, p. 176). In total, these secondary extensions resulted in the inclusion of 13 RCTs, four of which were designated "commercial-in-confidence" and not disclosed (TAR, Chapter 6).

In a final step, also the MTA study [25,26] – arguably the most important clinical study completed in ADHD to date – was added, although it remains enigmatic which data were actually used, as the model used information from three out of the four study arms only (TAR, p. 254): Whereas it was stated that "the nature of the treatment received in the community comparison arm of the MTA trial is still unclear, and as a result this data is omitted from the analysis" (TAR, p. 254), a table on the same page of the assessment report explicates that "results for behavioral treatment were omitted as not relevant to this review" (TAR, p. 254).

Whereas the assessment team did not explain its implicit assumption that CGI-I subscores – its primary measure of effectiveness used for the calculation of "response rates", which directly refers to a comparison "to the patient's condition before admission to the project" [38] – were independent from baseline, it rejected the Conners Rating Scales – the most widely used group of measurement instruments in ADHD studies [24] – for precisely this reason (TAR, p. 186 and p. 224). As a consequence, (apart from the enigmatic use of MTA study data) none of the 14 extended treatment studies reviewed by Schachar et al. (2002) [39] were included in data synthesis for cost-effectiveness evaluation (cf. TAR, Chapter 6). Also insights from an important 24-months RCT involving more than 100 patients treated with methylphenidate [40,41] were not considered because the study did not fit the narrowly defined inclusion criteria for review. Finally, discussion in the assessment report of the 24-months follow-up data from the MTA study, providing insights into the persistence of treatment effects over the first ten months after trial completion [42], was limited to the clinical review (TAR, p. 168); these data were not addressed in the context of the economic modeling exercise (TAR, Chapter 6).

Although the assessment group correctly observed that MTA subgroup analyses "should be seen as 'exploratory', because of the danger of repeated statistical testing with a sample not designed for this purpose" (TAR, p. 167), it claimed at the same time that its own "model is probabilistic, meaning that relevant input parameters are entered as probabilistic distributions in order to represent the uncertainty around each point estimate" (TAR, p. 220), emphasizing that "the output from the model incorporates the uncertainty around the estimated response rates" (TAR, p. 229). However, in RCTs it is the primary analysis, as defined *ex ante*, which is most important, and the CGI scores did not represent the primary endpoint in any of the studies selected for synthesis. While it is certainly legitimate to carry out secondary analyses, these should not be represented as fully capturing stochastic uncertainty [43].

So-derived differences in QALYs gained by each treatment "strategy" extended to the third or fourth decimal place only (TAR, pp. 237ff.), and the primary analysis produced a series of inconsistent rankings (TAR, p. 237), which were left uncommented and disappeared only after secondary model extensions ("sensitivity analyses", TAR, pp. 240ff.) comprising the pooling of heterogeneous response criteria mentioned earlier. Although it was claimed that "the issue of heterogeneity was overcome by basing the base case [primary] analysis on trials that are more similar in terms of how they measure the outcome of interest" (TAR, p. 266), in fact internally consistent model results were achieved after this pooling only, and there is no indication that potential confounding effects between treatment strategies and effect measures were assessed.

### Efficacy versus effectiveness

An important issue pervading the NICE assessment is the way the distinction between efficacy and effectiveness was (not) addressed. Whereas RCTs follow an explanatory orientation ("*Can *the intervention work?"), economic evaluations to be meaningful require a pragmatic orientation ("*Does *the intervention work?") [16,44,45]. Efficacy data collected during RCTs deliberately and necessarily exclude naturalistic effects associated with a routine clinical practice setting, while effectiveness may be influenced by a number of external factors, notably including poor treatment compliance. "Artificially enhanced compliance" in RCTs has come to be recognized as a major threat to their external validity [46]. This is a relevant aspect since the more expensive treatment options evaluated (atomoxetine and modified-release formulations of methylphenidate) differ from their comparators by their simplified dosage regimens, which may be expected to result in improved treatment adherence in practice settings.

There are multiple streams of evidence supporting this expectation. First, there is a statistically significant association between complexity of dosage regimens and treatment compliance [47]. Second, there are reasons to assume that treatment adherence of patients with ADHD may be impaired by disease-specific factors [48,49]. Third, due to their pharmacokinetic and pharmacodynamic properties, the behavioral effects of short-acting stimulants dissipate rapidly after three to four hours – making these drugs prototypical examples of non-forgiving compounds regarding noncompliance [50-55]. Fourth, three independent retrospective database studies consistently indicate higher treatment persistence rates for patients receiving long-acting stimulants compared to short-acting formulations [56-60]. Although analyses based on administrative data typically do not allow differential analysis of reasons for treatment discontinuation and may be distorted by patient selection bias, data showing a higher number of prior diagnoses and significantly lower rates of accidents, injuries, emergency room visits, and hospitalizations among those treated with long-acting formulations are consistent with the assumption that such distortions were absent [56,59,60]. Fifth, mediator analyses of the NIMH MTA study confirm the important role of compliance: as intended acceptance/attendance was found to significantly enhance treatment response for both the medication management and combined treatment strategies [26]. The NICE assessment group overlooked these findings (TAR, pp. 167ff.) and reasoned instead that "we can also incorporate the results of the MTA trial, but only by assuming that the medical management group in that trial represents treatment with immediate-release methylphenidate" (TAR, p. 253).

For economic evaluation, there are two broadly accepted ways to address the impact of compliance [61,62], i.e., the use of decision analytic models combined with appropriate sensitivity analyses and information from randomized "pragmatic trials" with minimal study management [63]. Modeling studies, sixth, have been indicative of an acceptable cost-effectiveness of long-acting methylphenidate, possibly reaching extending dominance over short-acting formulations [17,18,64]. Seventh, a randomized open-label study comparing long-acting with short-acting methylphenidate reported a number-needed-to-treat (NNT) of 3.6 to 4.8 to achieve one additional responder (depending on response criterion applied), consistently below the NNTs synthesized for assessment (TAR, pp. 226ff.) [11,65,66], although this particular study was impaired by the absence of teacher-reported outcome ratings.

None of these aspects are reflected in the NICE assessment. Instead it was "assumed that the trial data [referring to double-blind, double-dummy ADHD trials] adequately captures the effect of compliance on response to treatment" (TAR, p. 232). As a consequence, data from highly controlled double-blind RCTs and from randomized pragmatic open-label studies were pooled, necessarily concealing any differentiation on grounds of treatment compliance. Nevertheless it was claimed "the effect of compliance on response rates [...] is reflected in the model" (TAR, p. 250).

### Economic model

**Figure 1:**
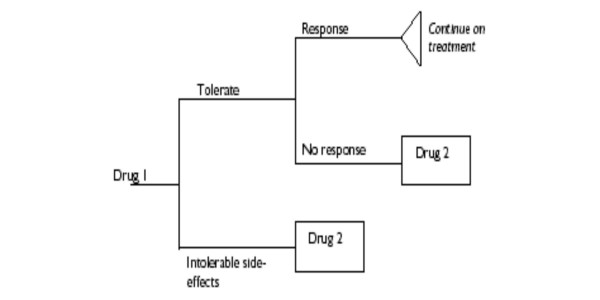
**Structure of the economic model**. The economic model was composed of modules for each product, which had a common structure and were combined sequentially to reflect "treatment strategies". The structure of the modules implied, inter alia, that the withdrawal rates "due to intolerable side-effects" should be independent from "no response" to treatment. This requirement was violated because intent-to-treat analyses were used to estimate withdrawal rates, which reported "withdrawals" for many reasons, including lack of efficacy, inevitably leading to double-counting of nonresponders (TAR, p. 230). The impact of this phenomenon was unevenly distributed across the treatments evaluated (TAR, p. 231 and p. 236), resulting in a biased assessment [11]. Graphical representation of model reproduced from King et al. 2006, with kind permission.

The economic model (cf. Fig. 1) relied on cost per QALY estimates based on response rates, using utility weights taken from one study reporting parent-proxy ratings using the EQ-5D [67] but not from standard gamble experiments as stated in the assessment report (TAR, p. 235). Although the structure of the model implied assumptions on withdrawal rates, which caused double-counting of nonresponders (TAR, p. 230), no attention was paid to the uneven effect this modeling approach had on the treatment options evaluated: the fact that for dexamphetamine extremely low withdrawal rates were assumed (TAR, p. 236, solely based on the study by Sharp et al., 1999 [30], with n = 32 girls observed over three weeks, which had initially been excluded: TAR, p. 231; cf. also TAR, pp. 225f. and p. 338) could only bias the modeled "treatment continuation rates" (cf. TAR, p. 222f.) in favor of dexamphetamine. This source of bias remained unmentioned.

None of the studies selected for the primary model exceeded an observation period of eight weeks per treatment arm (TAR, p. 226), and also the secondary model extensions (referred to as "sensitivity analyses") were informed by trials with observations periods of 12 weeks or less, except for the data extracted from three out of four parallel arms of the MTA study (TAR, p. 254). On this basis, costs and benefits were extrapolated over a time horizon of 12 years. Although the assessment includes a discussion of sensitivity of findings to time horizon (TAR, pp. 245ff.), this did not include a review of long-term sequelae associated with ADHD. No attempt was made to address the impact of the disorder on educational outcomes, injuries and accidents, or other problems such as encounters with the criminal justice system and the burden of caregivers.

The technology assessment purports to have "clearly identified an optimal treatment strategy" (TAR, pp. 19, 261, 266). The caveats offered essentially relate to a paucity of evidence and poor reporting of studies (e.g., TAR, pp. 18ff., 266), which were blamed for the inability to discriminate between drugs in efficacy or between patients in terms of ADHD subtype, age, gender or previous treatment (TAR, pp. 266f.).

## Conclusion

The NICE ADHD health technology assessment does not provide a complete account of the problem addressed. From an economic perspective, the omission from analysis of psychosocial interventions, representing a mainstay of ADHD therapy, is especially disturbing, as estimates of allocative efficiency require all possible options to be considered.

Furthermore the literature search was incomplete, existing evidence was used in an overly restrictive manner, and neither long-term sequelae nor caregiver burden were addressed. These shortcomings were confounded by outright technical errors, including but not limited to [11] the apparent confusion of efficacy and effectiveness.

Although the assessment may have its uses in listing existing literature and presenting condensed summaries, it leaves substantial room for improvement. Its main conclusions rest on shaky grounds and are potentially misleading. NICE itself, in its final appraisal determination and its guidance issued in March 2006, wisely moderated the putatively "clear conclusions" of the technology assessment report [3]. Understandably the appraisal process following the assessment was unable to overcome the limitations of the latter, which on the basis of its restricted dataset could not address the full range of questions specified in its scope (cf. Results, above).

Regarding the technology assessment process, the broad range of observed anomalies can be interpreted as symptoms, which may be indicative of specific underlying problems. In the present case, such underlying issues appear likely to have included an insufficient integration of clinical and economic perspectives, the extraordinarily high level of standardization of NICE technology appraisals, enforcing the computation of clinical outcomes as quality-adjusted life years and hereby requiring the clinical problem definition to fit to a preconceived solution, and the apparent absence of effective quality assurance systems [11]. Beyond avoiding certain technical errors, a more appropriate evaluation strategy might have made better use of available data on symptomatic improvement such as Conners Rating Scale scores, considered the impact of treatment nonadherence in ADHD, addressed clinical studies and meta-analyses indicating differences in effectiveness between stimulants and nonstimulants, reflected information on the importance of coexisting conditions and functional impairment, and discussed the long-term sequelae associated with ADHD [11].

While there remains a need for more research into the long-term effectiveness and cost-effectiveness of ADHD treatment strategies [68], at the same time a new, state-of-the-art systematic review including economic evaluations would be most welcome.

## Competing interests

There was no third-party or industry involvement in the present study, which was funded by the Institute for Innovation & Valuation in Health Care (InnoVal-HC). InnoVal-HC is a not-for-profit organization accepting support under a policy of unrestricted educational grants only. Potential competing interests: The Institute and/or its staff report having received public speaking and conference attendance as well as project support from payers', physicians', and pharmacists' associations, and served on advisory boards for companies including E. Lilly, Johnson & Johnson, Novartis, Pfizer, and Shire.

## References

[B1] Gilbody SM, Petticrew M (1999). Rational decision-making in mental health: the role of systematic reviews. Journal of Mental Health Policy and Economics.

[B2] World Health Organization (WHO) (2003). Technology appraisal programme of the National Institute for Clinical Excellence. A review by WHO. June-July 2003.

[B3] National Institute for Health and Clinical Excellenc (NICE) (2006). Methylphenidate, atomoxetine and dexamfetamine for attention deficit hyperactivity disorder (ADHD) in children and adolescents. Review of Technology Appraisal 13.

[B4] King S, Griffin S, Hodges Z, Weatherly H, Asseburg C, Richardson G, Golder S, Taylor E, Drummond M, Riemsma R (2004). A systematic review of the clinical and cost-effectiveness of methylphenidate hydrochloride, dexamfetamine sulphate and atomoxetine for attention deficit hyperactivity disorder (ADHD) in children and adolescents (commercial in confidence information removed). York.

[B5] Pearson SD, Rawlins MD (2005). Quality, innovation, and value for money. NICE and the British National Health Service. Journal of the American Medical Association (JAMA).

[B6] Centers for Disease Control and Prevention (CDC) (2005). Mental health in the United States. Prevalence of diagnosis and medication treatment for attention-deficit/hyperactivity disorder – United States, 2003. MMWR Morbidity and Mortality Weekly Report.

[B7] National Institute for Clinical Excellence (NICE) (2004). Guide to the Technology Appraisal Process (reference N0514).

[B8] National Institute for rClinical Excellence (NICE) (2004). Guide to the Methods of Technology Appraisal (reference N0515).

[B9] Daniels N, Sabin JE (2002). Setting Limits Fairly – Can We Learn to Share Medical Resources?.

[B10] Schlander M (2007). NICE accountability for reasonableness. A qualitative case study of its appraisal of treatments for attention-deficit/hyperactivity disorder (ADHD). Current Medical Research & Opinion.

[B11] Schlander M (2007). Health Technology Assessments by the National Institute for Health and Clinical Excellence (NICE): a case study of its recent appraisal of treatment strategies for attention-deficit/hyperactivity disorder.

[B12] King S, Griffin S, Hodges Z, Weatherly H, Asseburg C, Richardson G, Golder S, Taylor E, Drummond M, Riemsma R (2006). A systematic review and economic model of the effectiveness and cost-effectiveness of methylphenidate, dexamfetamine and atomoxetine for the treatment of attention deficit hyperactivity disorder in children and adolescents. Health Technology Assessment.

[B13] National Institute for Clinical Excellence (NICE) (2003). Health Technology Appraisal: Methylphenidate, atomoxetine and dexamfetamine for attention deficit hyperactivity disorder (ADHD) in children and adolescents including review of existing guidance number 13 (Guidance on the Use of Methylphenidate [Ritalin, Equasym] for Attention Deficit/Hyperactivity Disorder [ADHD] in childhood) – Scope.

[B14] King S, Riemsma R, Hodges Z, Emmany Dean M, Golder S, Drummond M, Weatherly H, Griffin S, Richardson G, Taylor E, Senn S (2004). Technology Assessment Report for the HTA Programme: Methylphenidate, dexamfetamine and atomoxetine for the treatment of attention deficit hyperactivity disorder. Final version.

[B15] Arnold LE, Jensen PJ, Cooper J (2002). Treatment alternatives for attention-deficit/hyperactivity disorder. Attention Deficit hyperactivity Disorder: State of the Science; Best Practices.

[B16] Centre for Reviews and Dissemination (CRD) (2001). CRD Report Number 4 Undertaking systematic reviews of research on effectiveness: CRD's guidance for those carrying out or commissioning reviews.

[B17] Annemans L, Ingham M (2000). Estimating cost-effectiveness of Concerta OROS in attention-deficit/hyperactivity disorder (ADHD) – adapting the Canadian Coordinating Office for Health Technology Assessment's (CCOHTA) economic model of methylphenidate immediate release versus behavioural interventions from a parent's perspective. Value in Health.

[B18] Schlander M (2004). Cost-effectiveness of methylphenidate OROS for attention-deficit/hyperactivity disorder (ADHD): an evaluation from the perspective of the UK National Health Service (NHS). Value in Health.

[B19] Jensen PS, Garcia JA, Glied S, Foster EM, Schlander M, the MTA Cooperative Group (2004). Cost-effectiveness of attention-deficit/hyperactivity disorder (ADHD) treatments: estimates based upon the MTA study. 16th World Congress of the International Association for Child and Adolescent Psychiatry and Allied Professions (IACAPAP).

[B20] Jensen PS, Garcia JA, Glied S, Crowe M, Foster M, Schlander M, Hinshaw S, Vitiello B, Arnold LE, Elliott G, Hechtman L, Newcorn JH, Pelham WE, Swanson J, Wells K (2005). Cost-effectiveness of ADHD treatments: findings from the multimodal treatment study of children with ADHD. American Journal of Psychiatry.

[B21] Michelson D, Faries D, Wernicke J, Kelsey D, Kendrick K, Sallee FR, Spencer T, Atomoxetine ADHD Study Group (2001). Atomoxetine in the treatment of children and adolescents with attention-deficit/hyperactivity disorder: a randomized, placebo-controlled, dose response study. Pediatrics.

[B22] Newcorn JH, Owens JA, Jasinski DR Results from recently completed comparator studies with atomoxetine and methylphenidate. 51st Annual Meeting of the American Academy of Child & Adolescent Psychiatry (AACAP).

[B23] American Psychiatric Association (APA) (2000). Handbook of Psychiatric Measures.

[B24] Collett BR, Ohan JL, Myers KM (2003). Ten-year review of rating scales. V. Scales assessing attention-deficit/hyperactivity disorder. Journal of the American Academy for Child and Adolescent Psychiatry.

[B25] MTA Cooperative Group (1999). A 14-month randomized clinical trial of treatment strategies for attention-deficit/hyperactivity disorder. Archives of General Psychiatry.

[B26] MTA Cooperative Group (1999). Moderators and mediators of treatment response for children with attention-deficit/hyperactivity disorder: the multimodal treatment study of children with attention-deficit/hyperactivity disorder. Archives of General Psychiatry.

[B27] Arnold LE (1996). Sex differences in ADHD: conference summary. Journal of Abnormal Child Psychology.

[B28] Sonuga-Barke EJS, Coghill D, Markowitz JS, Swanson JM, Vandenberghe M, Hatch SJ (2007). Sex differences in the response of children with ADHD to once-daily formulations of methylphenidate. Journal of the American Academy of Child and Adolescent Psychiatry.

[B29] Faraone SV, Sergeant J, Gillberg C, Biederman J (2003). The worldwide prevalence of ADHD: is it an American condition?. World Psychiatry.

[B30] Sharp WS, Alter JM, Marsh WL, Ritchie GF, Hamburger SD, Castellanos FX (1999). ADHD in girls: clinical comparability of a research sample. Journal of the American Academy of Child & Adolescent Psychiatry.

[B31] Newcorn J, Kratochvil CJ, Allen AJ, Milton DR, Moore RJ, Michelson M, Boca Raton FL Atomoxetine and OROS methylphenidate for the treatment of ADHD: acute results and methodological issues. Poster presentation at 45th Annual Meeting of the New Clinical Drug Evaluation Unit (NCDEU) of the National Institute of Mental Health (NIMH).

[B32] Kemner JE, Starr HL, Brown DL, Ciccone PE, Lynch JM Greater symptom improvement and response rates with OROS MPH vs atomoxetine in children with ADHD. XXIVth Congress of the Collegium Internationale Neuro-Psychopharmacologicum.

[B33] Kemner JE, Starr HL, Ciccone PE, Hooper-Wood CG, Crockett RS (2005). Outcomes of OROS methylphenidate compared with atomoxetine in children with ADHD: a multicenter, randomized prospective study. Advances in Therapy.

[B34] Steinhoff K, Wigal T, Swanson J, Miami FL Single daily dose ADHD medication effect size evaluation. 50th Annual Meeting of the American Academy for Child and Adolescent Psychiatry.

[B35] Faraone SV (2003). Understanding the effect size of ADHD medications: implications for clinical care. Medscape.

[B36] Faraone SV, Spencer T, Aleardi M (2006). Comparing the efficacy of medications used for ADHD using meta-analysis. MedGenMed.

[B37] Faraone SV, Biederman J, Spencer TJ, Aleardi M (2006). Comparing the efficacy of medications for ADHD using meta-analysis. Medscape General Medicine.

[B38] Guy W (1976). ECDEU Assessment Manual for Psychopharmacology – Revised (DHEW Publ No ADM 76–338).

[B39] Schachar R, Jadad AR, Gauld M, Boyle M, Booker L, Snider A, Kim M, Cunningham C (2002). Attention-deficit hyperactivity disorder: critical appraisal of extended treatment studies. Can J Psychiatry.

[B40] Klein RG, Abikoff HG, Hechtman L, Weiss G (2004). Design and rationale of controlled study of long-term methylphenidate and multimodal psychosocial treatment in children with ADHD. Journal of the American Academy of Child and Adolescent Psychiatry.

[B41] Abikoff HG, Hechtman L, Klein RG, Weiss G, Fleiss K, Etcovitch J, Cousins L, Greenfield B, Martin D, Pollack S (2004). Symptomatic improvement in children with ADHD treated with long-term methylphenidate and multimodal psychosocial treatment. Journal of the American Academy of Child and Adolescent Psychiatry.

[B42] MTA Cooperative Group (2004). National Institute of Mental Health Multimodal Treatment Study of ADHD follow-up: 24-month outcomes of treatment strategies for attention-deficit/hyperactivity disorder. Pediatrics.

[B43] Petitti DB (2000). Meta-Analysis, Decision Analysis, and Cost-Effectiveness Analysis Methods for Quantitative synthesis in Medicine.

[B44] Schwartz D, Lellouch J (1967). Explanatory and pragmatic attitudes in therapeutic trials. Journal of Chronic Diseases.

[B45] Weinstein MC, O'Brien B, Hornberger J, Jackson J, Johannesson M, McCabe C, Luce BR (2003). Principles of good practice for decision analytic modeling in health-care evaluation: report of the ISPOR Task Force on good research practices – modeling studies. Value in Health.

[B46] Ramsey S, Willke R, Briggs A, Brown R, Buxton M, Chawla A, Cook J, Glick H, Liljas B, Petitti D, Reed S (2005). Good research practices for cost-effectiveness analysis alongside clinical trials: the ISPOR RCT-CEA task force report. Value in Health.

[B47] Claxton A, Cramer J, Pierce C (2001). A systematic review of the associations between dose regimens and medication compliance. Clinical Therapeutics.

[B48] Hack S, Chow B (2001). Pediatric psychotropic medication compliance: a literature review and research-based suggestions for improving treatment compliance. Journal of Child and Adolescent Psychopharmacology.

[B49] Swanson J (2003). Compliance with stimulants for attention-deficit/hyperactivity disorder. Issues and approaches for improvement. CNS Drugs.

[B50] Swanson JM, Kinsbourne M, Roberts W, Zucker K (1978). A time-response analysis of the effect of stimulant medication on the learning ability of children referred for hyperactivity. Pediatrics.

[B51] Greenhill LL (1992). Pharmacologic treatment of attention deficit hyperactivity disorder. Psychiatric Clinics of North America.

[B52] Meredith PA, Métry J-M, Meyer UA (1999). Achieving and assessing therapeutic coverage. Drug Regimen Compliance: Issues in Clinical Trials and Patient Management.

[B53] Peck C, Métry J-M, Meyer UA (1999). Non-compliance and clinical trials: regulatory perspectives. Drug regimen compliance Issues in clinical trials and patient management.

[B54] American Academy of Child and Adolescent Psychiatry (AACAP) (2002). Practice parameter for the use of stimulant medications in the treatment of children, adolescents, and adults. J Am Acad Child Adolesc Psychiatry.

[B55] Greenhill LL, Perel JM, Rudolf G, Feldman B, Curran S, Puig-Antich J, Gardner R (2001). Correlations between motor persistence and plasma levels of methylphenidate-treated boys with ADHD. International Journal of Neuropsychopharmacology.

[B56] Lage M, Hwang P (2004). Effect of methylphenidate formulation for attention deficit hyperactivity disorder on patterns and outcomes of treatment. Journal of Child and Adolescent Psychopharmacology.

[B57] Marcus SC, Wan GJ, Kemner JE, Olfson M (2005). Continuity of methylphenidate treatment for attention-deficit/hyperactivity disorder. Archives of Pediatrics & Adolescent Medicine.

[B58] Sanchez RJ, Crismon ML, Barner JC, Bettinger T, Wilson JP (2005). Assessment of adherence measures with different stimulants among children and adolescents. Pharmacotherapy.

[B59] Kemner JE, Lage MJ (2006). Effect of methylphenidate formulation on treatment patterns and use of emergency room services. American Journal of Health System Pharmacy.

[B60] Kemner JE, Lage MJ (2006). Impact of methylphenidate formulation on treatment patterns and hospitalizations: a retrospective analysis. Annals of General Psychiatry.

[B61] Revicki DA, Frank L (1999). Pharmacoeconomic evaluations in the real world: effectiveness versus efficacy studies. Pharmacoeconomics.

[B62] Hughes DA, Bagust A, Haycox A, Walley T (2001). Accounting for noncompliance in pharmacoeconomic evaluations. Pharmacoeconomics.

[B63] March JS, Silva SG, Compton S, Shapiro S, Califf R, Krishnan R (2005). The case for practical clinical trials in psychiatry. American Journal of Psychiatry.

[B64] Schlander M Long-acting medications for the hyperkinetic disorders. A note on cost-effectiveness. Eur Child Adolesc Psychiatry.

[B65] Steele M, Riccardelli R, Binder C Effectiveness of OROS-methylphenidate vs. usual care with immediate release methylphenidate in ADHD children. American Psychiatric Association (APA) Annual Meeting, New York, NY.

[B66] Steele M, Weiss M, Swanson J, Wang J, Prinzo RS, Binder CE (2006). A randomized, controlled, effectiveness trial of OROS-methylphenidate compared to usual care with immediate-release-methylphenidate in Attention-Deficit-Hyperactivity-Disorder. Canadian Journal of Clinical Pharmacology.

[B67] Coghill D, Spender Q, Barton J, Hollis C, Yuen C, Cleemput I, Annemans L (2004). Measuring quality of life in children with attention-deficit/hyperactivity disorder in the UK. 16th World Congress of the International Association for Child and Adolescent Psychiatry and Allied Professions (IACAPAP).

[B68] Schlander M (2007). Impact of attention-deficit/hyperactivity disorder (ADHD) on prescription drug spending for children and adolescents: increasing relevance of health economic evidence. Child Adolesc Psychiatry Ment Health.

